# Research on Beampattern Synthesis of Conformal Array Based on Space-Variability Suppression

**DOI:** 10.3390/s24227239

**Published:** 2024-11-13

**Authors:** Sijia Liu, Minghai Pan

**Affiliations:** Key Laboratory of Radio Frequency Simulation and Radar Imaging, College of Electronic and Information Engineering, Nanjing University of Aeronautics and Astronautics, Nanjing 211100, China; panmh@nuaa.edu.cn

**Keywords:** conformal array, spatial variability, stable beam, weight optimization

## Abstract

In order to solve the problem that the array beampattern varies greatly with the angle during spatial scanning, this paper presents a synthesis method of the conformal array beampattern based on “spatial-variability (SV)” suppression. In order to obtain a stable beampattern, the convex optimization method is used to solve the weighted value of the amplitude and phase of the elements under the condition of satisfying the set beam gain threshold so that the optimized main lobes of the beams with different directions approach the main lobe of the reference beam. Since beams of different dimensions have different broadening degrees after optimization, we weighted the objective function and solved the optimal weights by the golden section search method (GSM) to improve the comprehensive optimization results of two-dimensional beams. The simulation results demonstrate the effectiveness of the proposed method.

## 1. Introduction

Array antennas are optimized by parameters such as the number of elements, array element position distribution, array element excitation amplitude, and phase to obtain the desired shaped beam, which is widely used in modern wireless electronic equipment [1]. Although planar array synthesis technology is relatively mature, the space physical structure determines that it is difficult to achieve stable wide-angle scanning. Due to the influence of the antenna unit pattern, the gain of the planar array is relatively low when the angle of the beam deviation normal is larger, and the radiation performance deteriorates rapidly with the increase in the beam scanning angle [2]. The conformal array can effectively reduce the cross-sectional area of radar scattering without changing the appearance of the structure of the carrier designed based on aerodynamic performance and has better stealth performance and combat capability [3]. At the same time, because the conformal array is not limited by the shape of the carrier, it can be conformal in the nose, fuselage, wing, tail, and other parts of the carrier, so the aperture of the array can be significantly expanded without increasing the number of array elements, and the full range of beam scanning coverage can be achieved.

For different conformal arrays, the author combines the traditional slot antenna conformal with the cylinder in ref. [4]. A new algorithm is proposed for estimating the direction of reach of the cylindrical conformal array based on directional antennas in ref. [5]. In ref. [6], the author studies the polarization MUSIC algorithm based on the cylindrical conformal array to improve the estimation accuracy of the arrival angle. Ref. [7] studies the beamforming algorithm based on a ring conformal array, and ref. [8] applies Capon beamforming to a semi-circular conformal array to improve the anti-interference ability. Ref. [9] studies the wide-angle beam scanning performance of complex conformal array antennas based on deep learning.

For conformal array beamforming, it is a very effective method to optimize the weighted values of the amplitude and phase of the counter elements to improve the beam performance. For scanning beamforming, the “space-variability (SV)” caused by the change in the scanning angle cannot be ignored, and it needs to be optimized to obtain a more stable beam performance. In ref. [10], the two-dimensional (2D) beam optimization problem of the conformal array is transformed into a convex optimization constraint problem, and the weights and array element distribution are optimized with the goal of high gain and low sidelobe, respectively. The convex optimization method is used to optimize the random array, and the minimum beamwidth under the current conditions is obtained on the premise of ensuring the maximum receiving power of the array in the specified direction in ref. [11]. The beamforming problem is transformed into a relaxation optimization problem to obtain the desired direction pattern by introducing a relaxation region and relaxation vector in ref. [12]. In ref. [13], a mathematical model of beamforming with constant beamwidth and constant direction under unconstrained conditions is established, which takes a certain direction as the reference main lobe and approximates the optimized beam main lobe with different directions to it. The authors of ref. [14] take main lobe-level constraints into account and use first-order Taylor expansion to deal with non-convex constraints with convex upper bound functions to achieve beamforming with minimum maximum sidelobe level. In ref. [15], a relaxation optimization problem is constructed to automatically determine the main lobe region and minimize the beamwidth in narrowband beampattern synthesis. In the convex optimization method, the threshold of constraint conditions should be set appropriately according to the prior information to avoid the unsolvable optimization problem.

In this paper, we derive the conformal array beam synthesis method and propose a beam optimization method to suppress the SV of the beampattern and finally obtain a gain- and shape-stable beampattern in the airspace. Firstly, we select the reference beam according to the demand of the scanning interval and set the number of elements participating in the beam under the set beam gain threshold. Then, in order to approximate the reference beam, the convex optimization method is used to optimize the amplitude–phase-weighted value of the element. When setting the optimization target, both the beam of azimuth section and pitch section are considered, the results of the two optimization targets are considered comprehensively by weighting the objective function, and the golden section search method (GSM) is used to solve the optimal weighted value.

The remainder of this paper is organized as follows: In Section 2, the conformal array beam synthesis method is explained. In Section 3, the optimization algorithm to suppress the SV of the beam is explained. In Section 4, the proposed algorithm is verified by simulation based on the truncated cone array. Finally, we provide conclusions in Section 5.

## 2. Conformal Array Beampattern Synthesis Method

In the conformal array, the distance between the elements is not uniform, and the position and normal direction of each element are different, so the representation of the beampattern of the conformal array is different from that of the uniform array. Let the conformal array have U normal elements pointing in different directions; the array receives the signal of the far-field target, and θ and φ, respectively, represent the pitch angle and azimuth angle of the incoming wave signal, that is, the angle corresponding to the position of the target. We need to calculate the pattern of each element, multiply it by the phase difference with the reference element, and finally accumulate it.

**Figure 1:**
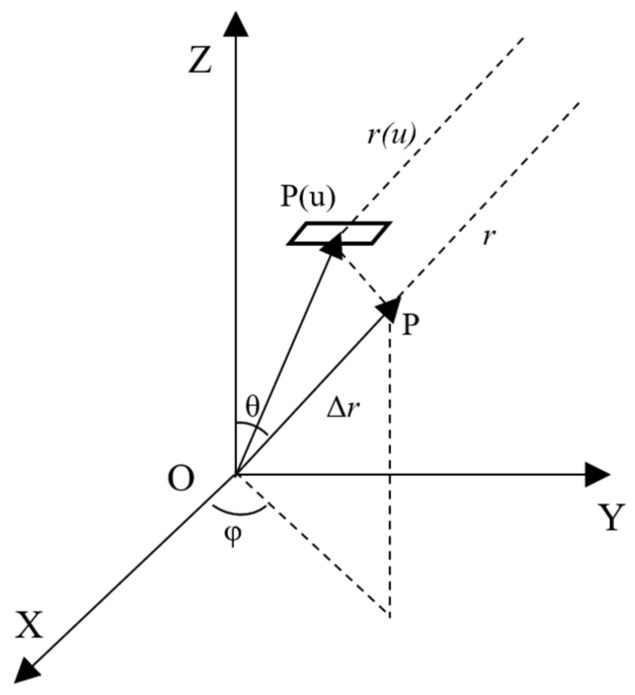
The wave path difference between the target P and its projection P (u) at element u.

Let the rectangular coordinates of the *u*-th element be expressed as (*x*(*u*), *y*(*u*), *z*(*u*)), and the range between each element and the radiation source after Taylor expansion and simplification can be expressed as (1):

(1) r−r(u)≈x(u)cosφsinθ+y(u)sinφsinθ+z(u)cosθ

where *r* is the range between the radiation source and the coordinate origin, and *r*(*u*) is the distance between each element and the coordinate origin. Figure 1 shows the wavelength difference in the element.

The phase difference between the target relative to the array element *Φ*(*u*) can be expressed as (2), where the wave number *k* = 2π/*λ*.

(2) ϕ(u)=k[r−r(u)]=k[(x(u)cosφsinθ+y(u)sinφsinθ+z(u)cosθ]

In the conformal array, due to the influence of carrier curvature, the position and normal direction of each element are different, and the element factor cannot be ignored relative to the element pattern. If the normal direction of the U-th subarray is (*φ*(*u*), *θ*(*u*)), the pattern *Fe*(*u*) of a single element can be calculated by (3a)~(3c), where *W* and *L* are the width and length of microstrip elements, respectively.

(3a) $$Fe\_\varphi (u)=\frac{\sin(\frac{kW\sin\varphi (u)\sin\theta (u)}{2})}{\frac{kW\varphi (u)\sin\theta (u)}{2}}\cos(\frac{kL}{2}\sin\theta (u)\cos\varphi (u))\cos\varphi (u)$$

(3b) $$Fe\_\theta (u)=\frac{\sin(\frac{kW\sin\varphi (u)\sin\theta (u)}{2})}{\frac{kW\sin\varphi (u)\sin\theta (u)}{2}}\cos(\frac{kL}{2}\sin\theta (u)\cos\varphi (u))\cos\theta (u)\sin\varphi (u)$$

(3c) $$Fe(u)=\sqrt{Fe\_\varphi {\left(u\right)}^{2}+Fe\_\theta {\left(u\right)}^{2}}$$

In order for the peak direction of the receiving beampattern to be (*φ*_0_, *θ*_0_), the phase shift value *Φ*_0_(*u*) provided by the phase shifter of the *u*-th element can be expressed as

(4) $$\phi_{0}(u)=k[r-r(u)]=k[x(u)\cos\varphi_{0}\sin\theta_{0}+y(u)\sin\varphi_{0}\sin\theta_{0}+z(u)\cos\theta_{0}]$$

The purpose of conformal array beamforming is to find the radiation field value of any point (*θ*, *φ*) in the array coordinate system, find the coordinates mapped to each element coordinate system (*θ*(*u*), *φ*(*u*)), then calculate these field values, and finally superposition the radiation field value at the point. Coordinate rotation transformation can be divided into three steps. That is, the polar coordinate system is first transformed to the rectangular coordinate system, then to the rectangular coordinate system of the element, and finally to the polar coordinate system of the element.

The amplitude and phase weighting value *ω*(*u*) is defined to satisfy *ω*(*u*) *= d*(*u*)*e*^(−*jφ*0(*u*))^; *d*(*u*) is the amplitude term. Then, the pattern of each element is calculated and multiplied by the phase difference with the reference element separately. Finally, we can obtain the beampattern pointing to (*φ*_0_, *θ*_0_) after superposition.

(5) $$\begin{array}{ll} F(\varphi ,\theta ) & =\sum_{u=1}^{U}d(u)\cdot Fe(u)\cdot \exp\{jk[(\cos\varphi \sin\theta -\cos\varphi_{0}\sin\theta_{0})x(u)+\\ & \left(\sin\varphi \sin\theta -\sin\varphi_{0}\sin\theta_{0})y(u)+(\cos\theta -\cos\theta_{0})z(u)]\right\} \end{array}$$

The default value of *d*(*u*) is 1. Equation (5) is rewritten as follows:

(6a) $$\begin{array}{ll} F(\varphi ,\theta ) & =\sum_{u=1}^{U}w(u)\cdot Fe(u)\cdot \exp\{jk[x(u)\cos\varphi \sin\theta +y(u)\sin\varphi \sin\theta +z(u)\cos\theta ]\}\\ & =w^{H}a(\varphi ,\theta ) \end{array}$$

where

(6b) $$w={\left[e^{-j\varphi_{0}(1)}\;e^{-j\varphi_{0}(2)}\;\cdots \;e^{-j\varphi_{0}(U)}\right]}^{T}$$

(6c) $$a(\varphi ,\theta )=[Fe(1)e^{j\varphi (2)}\;Fe(2)e^{j\varphi (2)}\;\cdots \;Fe(U)e^{j\varphi (U)}]$$

**Figure 2:**
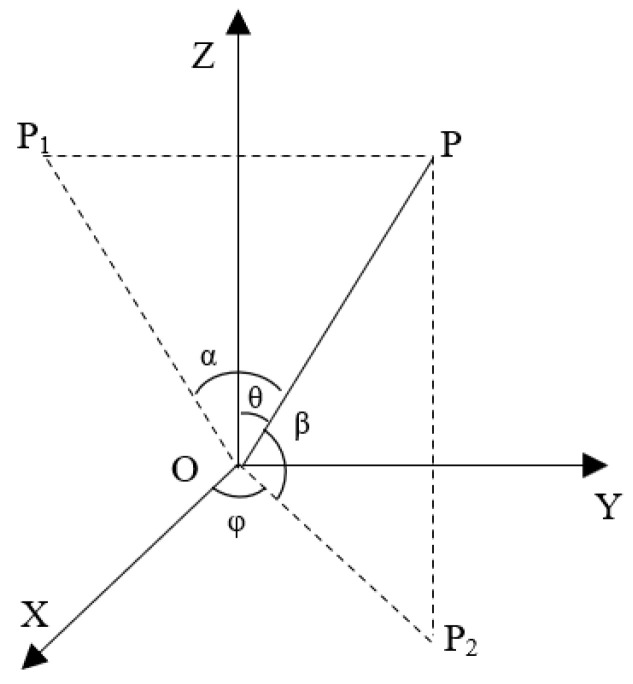
Azimuth–pitch coordinate system.

In forward-looking detection and imaging, we usually regard the forward direction of the platform as (0, 0), and the original polar coordinate system coordinate system cannot form a 2D beam when the pitch angle is 0°. Therefore, the azimuth–pitch coordinate system needs to be used, as shown in Figure 2.

In the azimuth–pitch coordinate system, the azimuth angle *α* is the angle between OP and XOZ plane, that is ∠POP_1_, and pitch angle *β* is the angle between OP and XOY plane, that is ∠POP_2_. The azimuth angle and pitch angle of the target in the azimuth–pitch coordinate system of the element are set as *α*(*u*) and *β*(*u*), respectively. Coordinate transformation is performed according to (7).

(7) $$\left\{\begin{array}{l} \alpha (u)=\arcsin(\sin(\varphi (u))\sin(\theta (u)))\\ \beta (u)=\pi /2-\theta (u) \end{array}\right.$$

Calculation is performed by substituting (7) into (6a). Finally, the beampattern in the azimuth–pitch coordinate system can be expressed as (8).

(8) $$\begin{array}{ll} F(\alpha ,\beta )= & \sum_{u=1}^{U}w(u)\cdot Fe(u)\cdot \exp\{jk[x(u)\cos\alpha \cos\beta +y(u)\sin\alpha +z(u)sin\beta ]\}\\ & =w^{H}a(\alpha ,\beta ) \end{array}$$

## 3. An Optimization Algorithm for Suppressing the “Spatial Variability” of the Beampattern

When the beam is scanned, the shape of the beampattern and the beam gain will change greatly with the beam direction. In order to ensure the performance of the array antenna for imaging and detection requirements, it is necessary to keep the beamwidth and beam gain basically unchanged, or the drop is not obvious in the scanning area. To solve the above problems, this section changes the beam shape by optimizing the amplitude and phase weighting value of the element so as to achieve the goal of suppressing the SV of the beamwidth and beam gain.

In general, the beam gain reflects the improvement in the Signal-to-Noise Ratio (SNR) brought about by the array, which is defined as the ratio of the output signal SNR of the array to the input signal SNR of the array. In the absence of interference and in an environment where each channel is considered to have the same noise spectrum, the noise covariance matrix evolves into a unit matrix. Therefore, the maximum array gain corresponding to the main beam direction (*α_i_*, *β_i_*) is defined in this section, as shown in (9), where *R_i_* is the guiding vector covariance matrix for the main beam direction.

(9) $$G(\alpha_{i},\beta_{i})=\frac{w^{H}a(\alpha_{i},\beta_{i})a{\left(\alpha_{i},\beta_{i}\right)}^{H}w}{w^{H}w}=\frac{w^{H}R_{i}w}{w^{H}w}$$

In order to achieve beam stabilization in the scanning interval, we can choose the direction diagram under a certain angle (*α*_0_, *β*_0_) as the reference direction diagram *F_ref_* according to the demand, and the result after translating to the current angle (*α_i_*, *β_i_*) is expressed as *F_refi_*. The main lobe region Ω_M_ and sidelobe region Ω_S_ are determined, and the constraints are the sidelobe region threshold ρ and gain threshold *G*_0_. In order to achieve the approximation between the current direction main lobe and the expected main lobe, and at the same time ensure convexity, we choose the *l_2_* norm minimization of the difference in the direction beampattern as the optimization objective. Before beam optimization, we can calculate the current beam gain *G_i_*. If *G_i_* < *G*_0_, the number of arrays can be increased before optimization. According to the above description, the problem to be solved can be expressed as (10).

(10a) $$\underset{w}{\min}\;\left\|w^{H}a(\alpha_{m},\beta_{m})-F_{refi}(\alpha_{m},\beta_{m})\right\|\;,(\alpha_{m},\beta_{m})\in \Omega_{M}$$

(10b) $$s.t.\left|w^{H}a(\alpha_{s},\beta_{s})\right|\leq \rho ,(\alpha_{s},\beta_{s})\in \Omega_{S}$$

(10c) $$\frac{w^{H}R_{i}w}{w^{H}w}\geq G_{0}$$

By analyzing the problem articulated by Equation (10), it becomes evident that the optimization objective requires point-by-point subtraction of the two beampatterns across the entire main lobe region. The substantial number of angle sampling points within this 2D main lobe region not only increases computational demands but also imposes greater constraints on the optimization objective, potentially influencing the optimization outcomes to some degree. Therefore, we can choose the azimuth and pitch dimension section beampattern corresponding to the current beam pointing angle as the optimization target so that the corresponding sections of the two one-dimensional beampatterns and the reference beampattern are approximated, and the two optimization problems are combined into one for consideration.

The main lobe region of the azimuth dimension is set to *Ω_Ma_*, and the main lobe region of the pitch dimension is set to *Ω_Mp_*. Because the change in *w* can change the beamwidth of the azimuth and pitch dimension at the same time, but the effect is not the same, the two optimization objectives are not independent. To solve this problem, we introduce the weighted value of the objective function *ζ* to adjust the weight of the optimization target, in order to obtain the optimal solution of the two optimization problems at the same time and obtain the comprehensive optimal 2D beam. To sum up, the optimization objective function is revised, as shown in (12a).

We find that the beam gain constraint (10c) is non-convex and cannot be solved directly by the cvx tool in Matlab 2016a. The main lobe peak level (ML) of the beam can also reflect the beam gain to a certain extent and can be easily obtained from the direction beampattern. According to the definition, the ML corresponding to the (*α_i_*, *β_i_*) can be expressed as (11).

(11) $$ML(\alpha_{i},\beta_{i})={\left|w^{H}a(\alpha_{i},\beta_{i})\right|}^{2}=\left|w^{H}a(\alpha_{i},\beta_{i})a{\left(\alpha_{i},\beta_{i}\right)}^{H}w\right|=\left|w^{H}R_{i}w\right|$$

Considering that the ML of the beam will change with the change in *w*, we choose to normalize the pattern with a fixed standard, rather than directly divide the ML of the current beam. When the change in *Fe*(*u*) is ignored and the default response mode value is 1, the corresponding ML is U^2^, so we divide by U^2^ to complete the normalization operation. According to the above analysis, we use the normalized ML constraint instead of the beam gain constraint and set the difference between the ML of the optimized pattern and the ML of the reference pattern to be less than *δ*, as shown in (12c).

Finally, the beam robustness constraint requires that the sum of array element excitations after optimization, that is, the total output power, is not larger than that before optimization. Since the excitation value of the normalized direction graph before optimization can be expressed as $\left|w_{0}\right|=\left|e^{-j\varphi_{0}}U^{-1/2}\right|=U^{-1/2}$, based on this, the norm of the weighted vector *w* is constrained, as shown in (12d).

(12a) $$\begin{array}{ll} \underset{w,\varsigma }{\min} & \;\varsigma \left\|w^{H}a(\alpha_{i},\beta_{p})-F_{refi}(\alpha_{i},\beta_{p})\right\|+(1-\varsigma )\left\|w^{H}a(\alpha_{a},\beta_{i})-F_{refi}(\alpha_{a},\beta_{i})\right\|,\\ & (\alpha_{a},\beta_{a})\in \Omega_{Ma},\;(\alpha_{p},\beta_{p})\in \Omega_{Mp} \end{array}$$

(12b) $$s.t.\left|w^{H}a(\alpha_{s},\beta_{s})\right|\leq \rho ,(\alpha_{s},\beta_{s})\in \Omega_{S}$$

(12c) $$\left|w^{H}a(\alpha_{i},\beta_{i})-F_{ref}(\alpha_{0},\beta_{0})\right|\leq \delta$$

(12d) $${\left\|w\right\|}^{2}\leq 1/U$$

Above, (12) is the optimization problem of the beampattern after adjustment. Compared with (10), the single optimization objective is replaced with two weighted objectives, and the optimization of the 2D beampattern is transformed into two profile beampatterns. The constraints are the sidelobe constraints, peak level constraints, and beam robustness constraints. The above constraints obviously conform to the structure of the convex problem, so the cvx tool can be used to solve it directly. When solving the above optimization problems, it is necessary to further analyze the values of some threshold parameters and the optimization results. For example, ρ can be set on demand, and *δ* can be determined based on prior information from the beampattern of the current angle, while ζ needs to be adjusted after the evaluation of the optimization results, and how to determine ζ will be analyzed below.

Under the condition that the number of array elements and the total power of array elements remain unchanged, the beam broadening will inevitably decrease with the ML. The value of *ζ* represents the focus of the other dimension or pitch dimension beam optimization results, and there must be a value ζ_0_ in the optimization process so that the optimization results of the 2D pattern can be optimized comprehensively under the condition that the beam gain threshold is satisfied.

**Figure 3:**
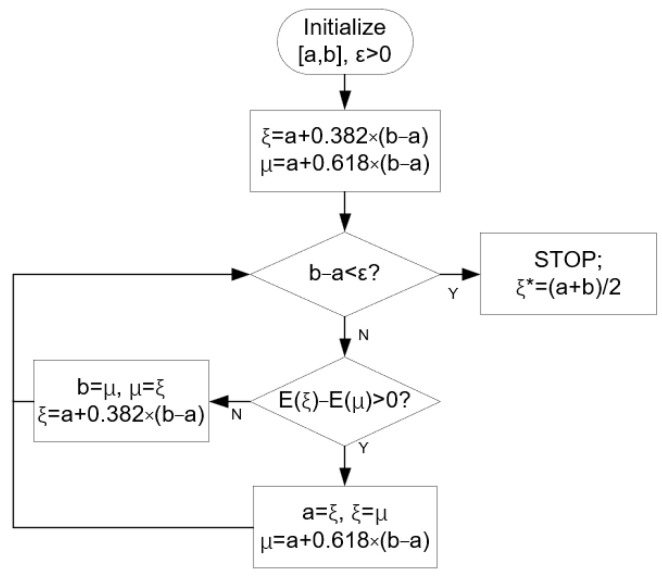
GSM algorithm flow.

The search method is used to solve the above problems. In order to speed up the process of interval contraction and search, we use the GSM to search for the *ζ* value corresponding to the optimal solution. First, two initial points *ξ*, *μ*∈[a, b] are determined according to the golden section value. If the interval size is greater than the set threshold ε, then *ξ*, *μ*, and [a, b] are updated according to the sizes of the evaluation functions E(ξ) and E(μ) in the iteration until the interval size is shrunk to the set threshold, and the middle point of the interval ξ* is taken as the result. The flow of the algorithm is shown in Figure 3.

Using this search algorithm, the corresponding solution with a smaller objective function can be quickly searched in a certain interval, which corresponds to the beampattern optimization problem to be solved in this section. The beamwidth variation error (BVE) is defined to compare the 2D beamwidth of the current beam and the desired beam, as shown in (13).

(13) $$BVE(\alpha_{i},\beta_{i})=\left|BW_{\beta }(\alpha_{i})-BW_{\beta }(\alpha_{0})\right|+\left|BW_{\alpha }(\beta_{i})-BW_{\alpha }(\beta_{0})\right|$$

where *BW_α_*() and *BW_β_*() represent the azimuth beamwidth and pitch beamwidth at a certain angle, respectively.

In addition, the pattern variation error (PVE) is defined to reflect the difference in the main lobe between the current beampattern and the expected one, as shown in (14).

(14) $$\begin{array}{ll} PVE(\alpha_{i},\beta_{i}) & =\left|\left|w^{H}a(\alpha_{i},\beta_{p})\right|-\left|F_{refi}(\alpha_{i},\beta_{p})\right|\right|+\left|\left|w^{H}a(\alpha_{a},\beta_{i})\right|-\left|F_{refi}(\alpha_{a},\beta_{i})\right|\right|,\\ & (\alpha_{a},\beta_{a})\in \Omega_{Ma},\;(\alpha_{p},\beta_{p})\in \Omega_{Mp} \end{array}$$

Obviously, Equations (13) and (14) do not satisfy convexity, so in the loop operation, we only use them as an evaluation of the optimization result. The beam width and deformation degree of the beam can be comprehensively considered according to the needs, and the evaluation function E(ζ) = *PVE*(ζ) + *BVE*(ζ) can be solved to minimize the ζ value of E(ζ) so as to obtain the best beam optimization results.

Finally, the beam optimization algorithm based on SV suppression, that is, the solving process of the beam optimizing problem (12), proposed in this section is summarized in Table 1.

## 4. Simulation Results

In this section, the conformal array beamforming and optimization methods proposed in the previous section are verified and explained by using the truncated cone array as a model. Firstly, we analyze the SV of the 2D beam, and then the proposed beam optimization algorithm is simulated and discussed under different ML thresholds and different angles to verify its effectiveness in suppressing the SV. Next, the weight search algorithm is simulated. Finally, the proposed algorithm is compared with similar optimization algorithms.

### 4.1. Simulation Condition

**Figure 4:**
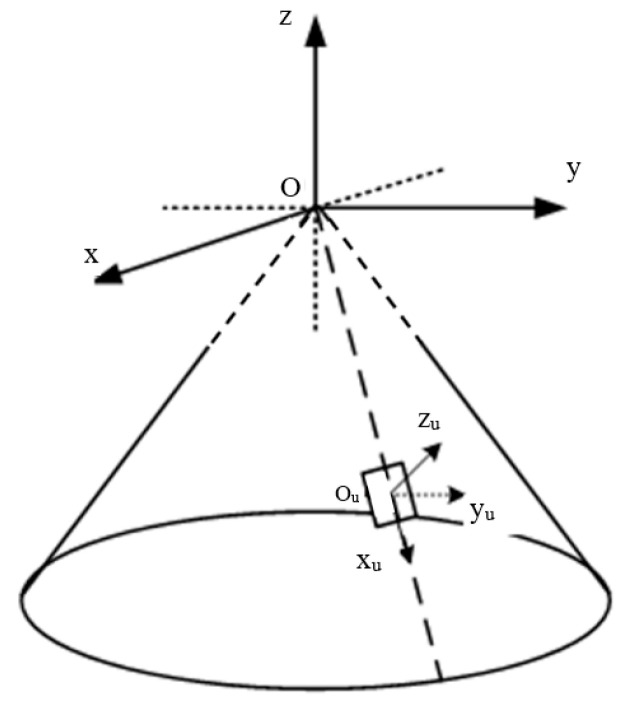
The truncated cone array structure.

**Figure 5:**
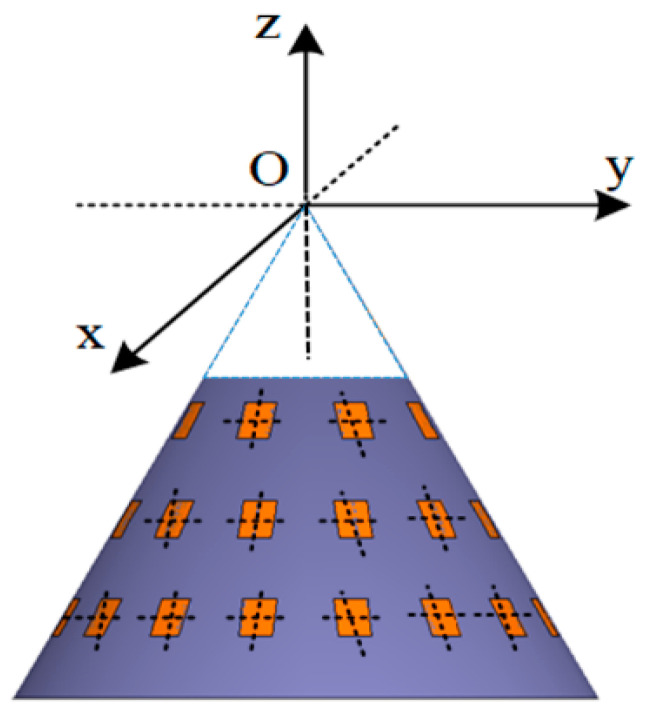
The element coordinate system.

The truncated cone array is symmetrical about the rotating axis, and the structure diagram is shown in Figure 4. The phase center of the element is located on the surface of the platform carrier, the x_u_oy_u_ plane of the local coordinate system is located on the tangent plane of the carrier, and the z_u_ axis is perpendicular to the tangent plane and points outward. The establishment of the element coordinate system is shown in Figure 5.

The number of elements per layer M_1_ = 75, the number of layers M_z_ = 20, and the total number of elements U = M_1_ × M_z_. The radius of the top surface R = 0.3 m, and the angle between the generatrix of the cone and the negative Z axis α_0_ = π/4.

### 4.2. Simulation of the Algorithm for Suppressing 2D Beam Spatial Variability

**Figure 6:**
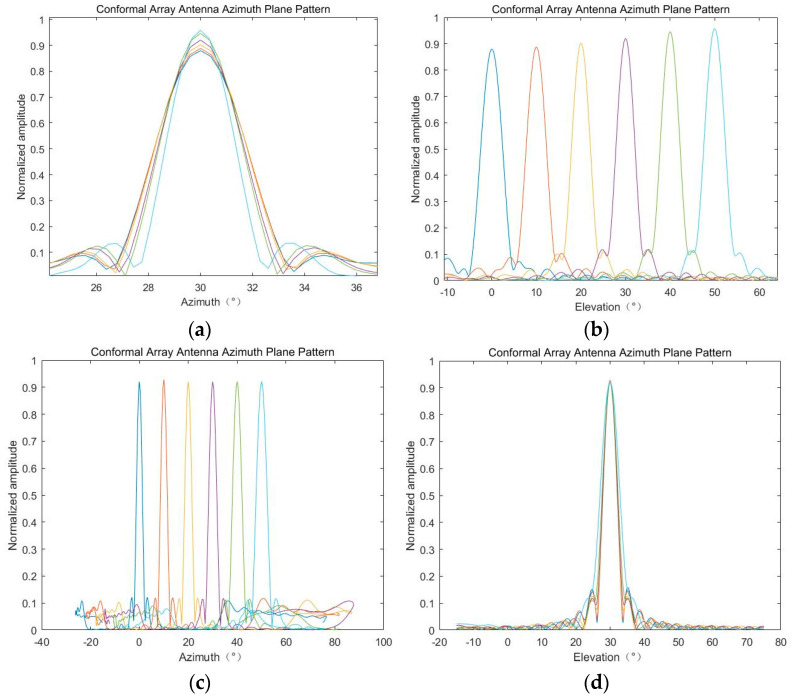
The SV of 2D beampattern: (**a**) α = 30°, β = 0~50°, azimuth pattern; (**b**) α = 30°, β = 0~50°, pitch pattern; (**c**) α = 0~50°, β = 30°, azimuth pattern; (**d**) α = 0~50°, β = 30°, pitch pattern.

First of all, we carry out simulation to verify the null variability of the 2D beam. We set the azimuth angle to be fixed at 30°, and the pitch angles are sampled at intervals of 10° within [0, 50] to obtain the 2D beampattern; then, we exchange the angle values and recalculate. The results corresponding to different angles are shown in Figure 6.

As can be seen from Figure 6, under the condition that the azimuth is fixed and only the pitch angle is changed, both the normalized ML and beamwidth of the 2D beampattern will change, and the situation is similar in the other case. In order to keep the beam gain and beam width stable during scanning, we select a suitable beampattern as a reference to optimize other beams during scanning.

**Figure 7:**
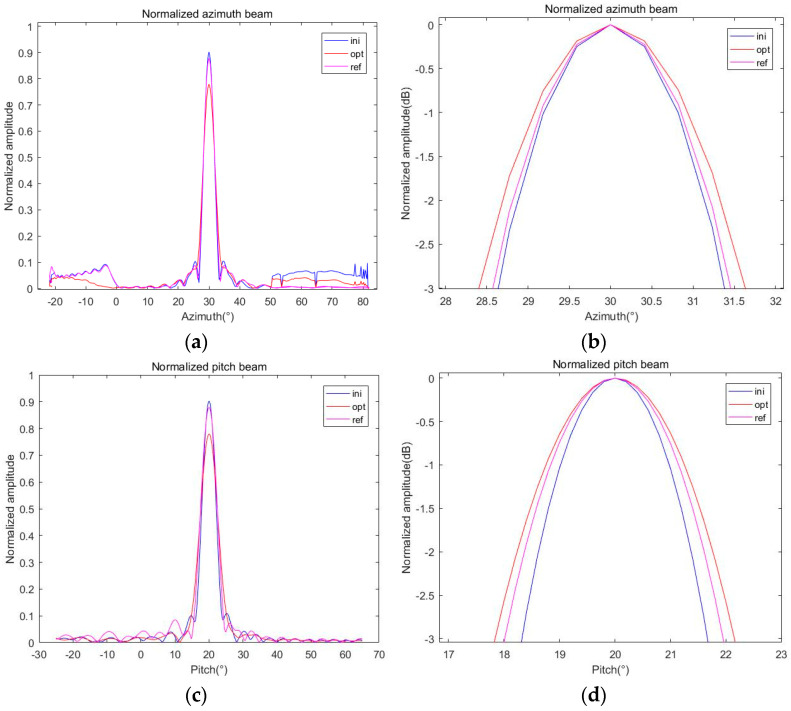
Initialized, optimized, and referenced 2D beampatterns under δ = 0.1 at (30, 20): (**a**) normalized azimuth beam; (**b**) normalized azimuth beam in dB; (**c**) normalized pitch beam; (**d**) normalized pitch beam in dB.

We select the reference angle (*α*_0_, *β*_0_) = (30, 0) and optimize the directional graph with the current angle (*α_i_*, *β_i_*) = (30, 20). Then, we set *G*_0_ = 24 dB, δ = 0.1, and ρ = 0.1 to solve the beam optimization problem of (12). The normalized initialized beampattern, optimized beampattern, and referenced beampattern are drawn in Figure 7a,c. In addition, in order to compare the difference in beam widths, several beampatterns are normalized by dividing the ML of the current beam and converted into dB units, as shown in Figure 7b,d.

It can be seen from the comparison of the beampatterns in Figure 7 that after optimization, the ML decreases by about 0.1, and the beampatterns in the azimuth direction and the pitch direction are broadened to a certain extent. Figure 7b shows that the broadening degree of the azimuth direction is larger, while the BVE in Figure 7d is smaller than the one before optimization. Therefore, we need to evaluate the optimization results and calculate the beam gain, PVE, and BVE, which are calculated according to Equations (9), (13), and (14), respectively, and summarized in Table 2 for comparison.

**Figure 8:**
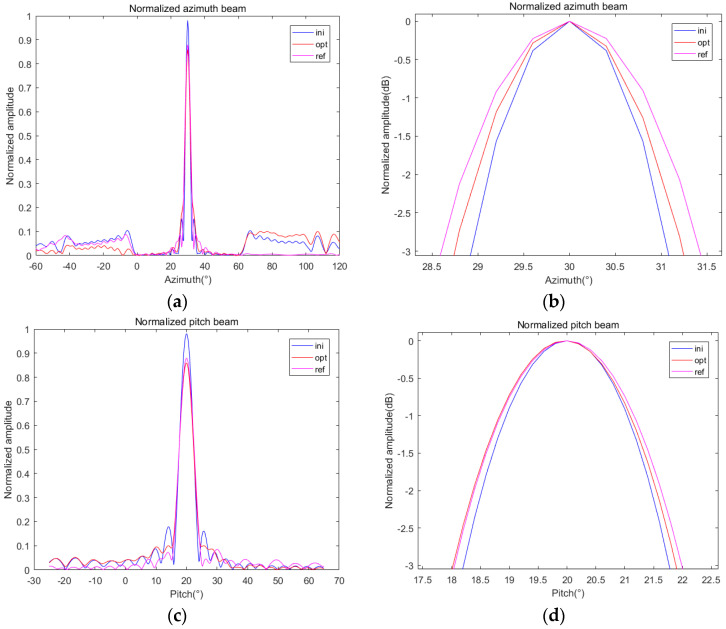
Initialized, optimized, and referenced 2D beampatterns under δ = 0.02 at (30, 20): (**a**) normalized azimuth beam; (**b**) normalized azimuth beam in dB; (**c**) normalized pitch beam; (**d**) normalized pitch beam in dB.

Keeping other conditions constant and adjusting *δ* to 0.05, Equation (12) is solved again, and the beampatterns are drawn like above; the result is shown in Figure 8.

As can be seen from Figure 8, the optimization problem (12) can also be solved after increasing *δ*, and the corresponding gain is improved. Compared with Figure 7, the broadening degree of the pattern is reduced, and the BVE is significantly reduced compared with that before optimization, and the optimization effect is obviously better than *δ* = 0.1. As before, relevant parameters are calculated and summarized in Table 2.

In order to reflect the universality of the algorithm, we change the target angle to (30, 45), keep *δ* = 0.02 unchanged, and then solve the optimization problem (12).

**Figure 9:**
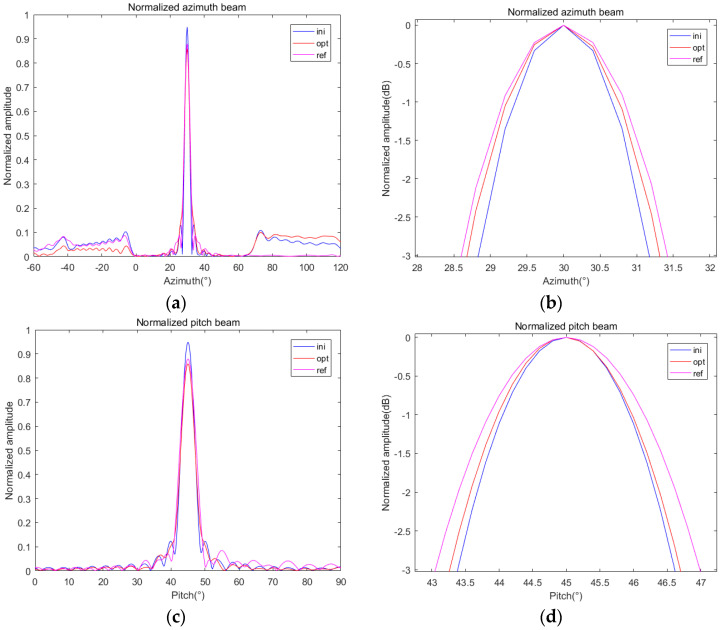
Initialized, optimized, and referenced 2D beampatterns under δ = 0.02 at (30, 45): (**a**) normalized azimuth beam; (**b**) normalized azimuth beam in dB unit; (**c**) normalized pitch beam; (**d**) normalized pitch beam in dB unit.

It can be seen from Figure 9 that the results are similar to (30, 20): the ML drops less than before, and the profile beampattern of the azimuth dimension and pitch dimension is closer to the expected beampattern. The relevant parameters are shown in Table 2.

As can be seen from Table 2, comparing the optimization results corresponding to different values at angles (30, 20), when δ = 0.1, although the pitch beamwidth is optimized to a certain extent, the decrease in the ML and the broadening degree of the azimuth pattern are too large, inevitably accompanied by a decrease in the beam gain and an increase in the PVE and BVE, leading to a larger difference than before. With the decrease in *δ*, the broadening range is reduced, and the beam gain is increased. Compared with the optimization results of (30, 45) and (30, 20), the PVE and BVE are significantly reduced compared with before optimization, that is, they are closer to the reference beampattern. Therefore, the algorithm proposed in this paper can optimize the beampattern in each direction into a stable beam with a gain and beamwidth close to the reference beampattern within a scanning interval and suppress the SV effectively, in order to meet the requirements of scanning detection and imaging.

It can be seen that under the current array, the pitch beamwidth is broadened more than the azimuth beamwidth. In order to balance the two optimization objectives better, we will conduct optimization after weighting the optimization objectives in the next section and solve the optimal weight ζ.

### 4.3. Simulation of Weighted Target Optimization and GSM Method

In this section, we further optimize the beam and use the GSM to search for the optimal weight ζ. Since the case without the weighted value is considered in the previous section, it can be considered that the optimization target weights of the azimuth dimension and pitch dimension are the same, so the initial weight ζ = 0.5.

**Figure 10:**
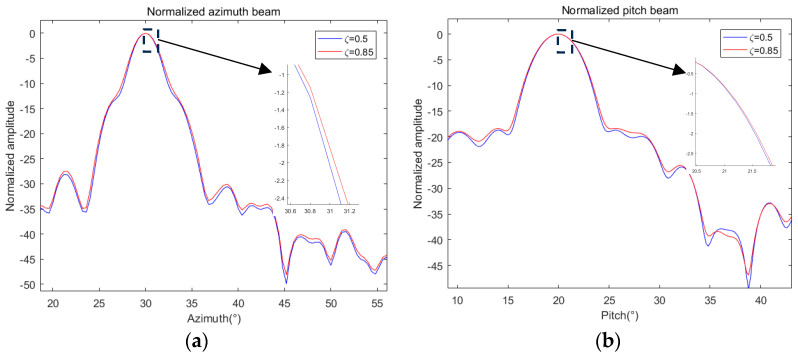
Comparison of optimization results of different ζ under (30, 20): (**a**) normalized azimuth beam; (**b**) normalized pitch beam.

Keeping other conditions unchanged, using the GSM shown in Table 1, we search for the optimal value of ζ. The minimum value of E(ζ) is obtained when ζ = 0.85. The optimization results are shown in Figure 10. 

It can be seen that after re-optimization, the pitch beamwidth has no significant change, the azimuth beamwidth is slightly increased, and the difference between the beamwidth and the reference beamwidth is narrowed, so the optimization result is slightly improved.

**Figure 11:**
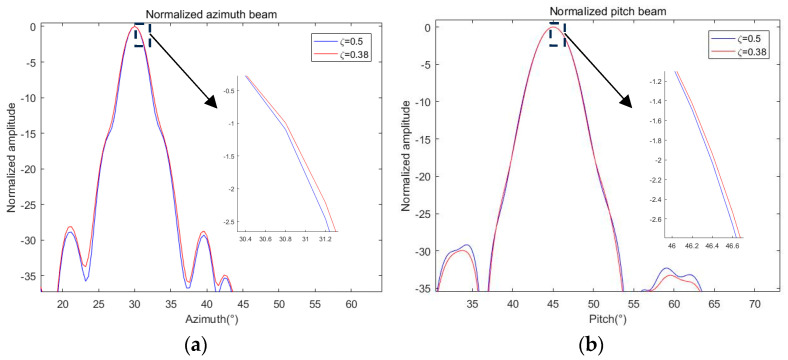
Comparison of optimization results of different ζ under (30, 45): (**a**) normalized azimuth beam; (**b**) normalized pitch beam.

In order to reflect the generality of the algorithm, we change the target angle to (30, 45) and use the GSM to solve ζ = 0.38 as the optimal value. Similarly, we simulate and compare the optimization results, as shown in Figure 11. 

Similar to the previous part, we summarized the simulation and calculation results of the above corresponding parameters in Table 3 for a more intuitive comparison.

It can be seen from Table 3 that after applying the optimal ζ obtained by the GSM to the objective function, both the PVE and BVE terms are reduced, indicating that the optimized beam is closer to the reference beam, so the optimization result is improved compared with the initial weight. The validity of the weighted optimization and GSM is proved.

### 4.4. Algorithm Performance Comparison

Finally, we compare the optimization of similar algorithms, selecting an algorithm in the literature [10] and the algorithm extended in ref. [14] to compare with the algorithm proposed in this paper. Both refs. [10,14] used convex optimization to complete the main optimization steps. The optimization object of ref. [10] is a 2D beampattern on a conformal array for the purpose of preserving the maximum beam gain. In ref. [14], a relaxation variable is introduced to broaden the beam width to reach the threshold value, and the optimization is carried out together with the amplitude and phase weight. Since ref. [14] is mainly carried out on a one-dimensional beampattern of a linear array, and the optimization focus is not exactly the same as the objective of this paper, we extend part of the method of ref. [14] to two-dimensional as a reference. The characteristics of the three algorithms are shown in Table 4.

**Figure 12:**
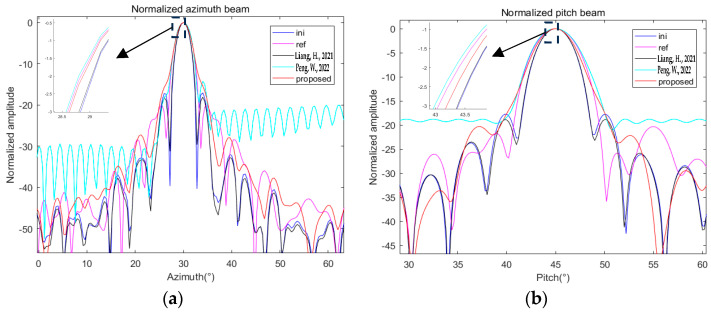
Comparison of optimization results of different algorithms under (30, 45): (**a**) normalized azimuth beam; (**b**) normalized pitch beam [10,14].

The simulation conditions remain the same as in the previous section. The three algorithms are used to optimize the beams generated based on the same truncated cone array model, and the results are shown in Figure 12.

Similarly, we also calculate the corresponding parameters and show them in Table 5.

Compared with the optimization results, the algorithm in ref. [10] has a higher beam gain, but because there is no constraint on the difference in the beampattern, the beamwidth is not much different from that before optimization, which can also be proved by the calculation results of the PVE and BVE. The results of the proposed algorithm and the extended method of ref. [14] are closer to the reference beampattern, and the optimization effect is basically the same. However, since the two relaxation variable sequences introduced need to be convex-optimized together with the amplitude–phase weights, the computational load of the solution is greatly increased, and the running time of the extended method of ref. [14] is significantly higher than that of the proposed algorithm. The above comparison shows that the optimization algorithm in this paper not only has good performance but can also introduce fewer extra parameters in solving optimization problems, making the algorithm complexity not too high.

## 5. Conclusions

In order to solve the problem that the conformal array needs a more stable beam in a wide range of scanning angles, this paper determines the criterion of controlling beam gain as a wide range of beamforming and proposes an optimization algorithm based on this. The algorithm aims to optimize 2D beampatterns simultaneously and is constrained by the main lobe peak level and sidelobe level. At the same time, we introduce the weighted value of the objective function to adjust the weights of different optimization problems and comprehensively consider the optimization results of 2D beampatterns and the GSM algorithm to search for and solve the weighted value. The simulation results show that the beampattern of the selected angle can be approximated to the referenced one after optimization under the set requirements. After the objective-function-weighted optimization, the result is further improved, and the SV can be suppressed effectively. In addition, compared with similar optimization algorithms, the proposed algorithm can take into account both the beam gain and beam width to obtain a stable beam and control the computation amount at the same time.

## Figures and Tables

**Table 1:** Summary of the proposed method for pattern synthesis.

Input	(*α_i_*, *β_i_*), [a, b], *G*_0_, *ρ*, *F_refi_*
1	Calculate beampattern by (8) and beam gain *G_i_* by (9) If *G_i_ < G*_0_, add the number of elements and repeat step 1
2	Determine *δ*, and initialize ζ_k_
3	while b − a > ε
4	k = k + 1
5	Substitute ζ_k_ into (12) and get *w_k_*
6	Calculate beampattern by (8), beam gain *G_i_* by (9) and evaluation function E(k)
7	if E(k) > E(k − 1), break;
8	Adjust search interval [a, b], Update search variable *ξ_k_*, *μ_k_*, and weight *ζ_k_*
9	end
10	end
11	Compute *ζ* = (*ξ_k_* + *μ_k_*)/2, substitute in (12) and get *w_opt*
**Output**	*w_opt*, *ζ*

**Table 2:** Comparison of optimization results under different *δ* and angles.

Angle	(30, 0)	(30, 20)			(30, 45)	
Condition	ref	ini	δ = 0.1	δ = 0.02	ini	δ = 0.02
Width_Azi	2.79	2.14	2.93	2.45	2.34	2.64
Width_Ele	3.90	3.58	4.17	3.95	3.23	3.45
Beam gain	24.68	25.03	23.63	24.48	25.13	24.48
PVE	-	2.5	2.5	1.08	1.77	1.51
BVE	-	0.96	0.40	0.40	1.12	0.58

**Table 3:** Comparison of optimization results under different ζ and angles.

Angle	(30, 0)	(30, 20)			(30, 45)		
Condition	ref	ini	ζ = 0.5	ζ = 0.85	ini	ζ = 0.5	ζ = 0.38
Width_Azi	2.79	2.14	2.45	2.62	2.34	2.64	2.75
Width_Ele	3.90	3.58	3.95	4.04	3.23	3.45	3.61
PVE	-	2.5	1.08	1.22	1.77	1.51	1.28
BVE	-	0.96	0.40	0.32	1.12	0.58	0.33

**Table 4:** Comparison of the characteristics of different algorithms.

Algorithm	[10]	Extended [14]	The Proposed Algorithm
Optimization objective	Beam gain	Beamwidth	Beamwidth
Extra parameter	None	Two sequences	One
Solving problems	One	Two	Two

**Table 5:** Comparison of optimization results of different algorithms.

Algorithm	ref	ini	[10]	Extended [14]	Proposed
Width_Azi	2.79	2.34	2.40	2.84	2.75
Width_Ele	3.90	3.23	3.26	4.00	3.61
Gain	24.68	25.13	25.20	24.20	24.48
PVE	-	1.77	1.80	1.30	1.28
BVE	-	1.12	1.04	0.15	0.33

## Data Availability

No new data were created or analyzed in this study. Data sharing is not applicable to this article.
